# Synchronous p16^+^ nasopharyngeal and oropharyngeal squamous cell carcinoma: a case report and review

**DOI:** 10.1002/ccr3.3765

**Published:** 2021-01-22

**Authors:** Muhammad Ali, Hock Kua, Charles Giddings, Daphne Day, Lachlan McDowell

**Affiliations:** ^1^ Department of Radiation Oncology Peter MacCallum Cancer Centre Melbourne Vic. Australia; ^2^ Department of Pathology Monash Health Melbourne Vic. Australia; ^3^ Department of Head and Neck Surgery Monash Health Melbourne Vic. Australia; ^4^ Department of Medical Oncology Monash Health Melbourne Vic. Australia; ^5^ Faculty of Medicine Monash University Melbourne Vic. Australia; ^6^ Sir Peter MacCallum Department of Oncology The University of Melbourne Melbourne Vic. Australia

**Keywords:** base of tongue, nasopharynx, p16^+^, squamous cell carcinoma, synchronous

## Abstract

We report a case of synchronous p16+ SCC involving both the nasopharynx and base of tongue treated with definitive chemo‐radiotherapy with concurrent high dose cisplatin. The nasopharyngeal lesion was detected incidentally on PET/CT imaging. Head and neck clinicians treating p16+ SCC should consider the possibility of synchronous lesions, including lesions which may be located in the lymphoid tissue of the nasopharynx.

## Graphical abstract

ET/CT suggested a likely occult lesion in the nasopharynx and adenoidectomy was required for confirmation of p16+ nasopharyngeal carcinoma.

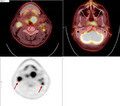

## 1 INTRODUCTION

Globally, the overwhelming majority of nasopharyngeal carcinomas (NPC) occur within endemic or migrant south East Asian populations driven by exposure to the Epstein‐Barr virus (EBV). However, human papillomavirus (HPV)‐associated NPC has also been described, mostly in Caucasian populations. Co‐infections with both EBV and HPV have been rarely reported and similar to oropharyngeal cancer (OPC), p16 shows high sensitivity and specificity for HPV‐associated NPC.^1^

There are few reports of synchronous or metachronous p16^+^/HPV^+^ cancers where the nasopharynx is primarily involved (excluding those with extension from the oropharynx). Caley et al^2^ reported 11 cases of synchronous and metachronous p16^+^ head and neck squamous cell carcinoma (SCC), which included two cases involving metachronous nasopharynx and OPC. McGovern el al described an interesting report of synchronous HPV‐associated SCC involving the bilateral tonsils and nasopharynx treated with radical intent radiotherapy and concurrent chemotherapy.^3^

We report a case of a patient presenting with synchronous p16^+^ SCC involving the nasopharynx and BOT treated at our institution

## 2 CASE REPORT

**FIGURE 1:**
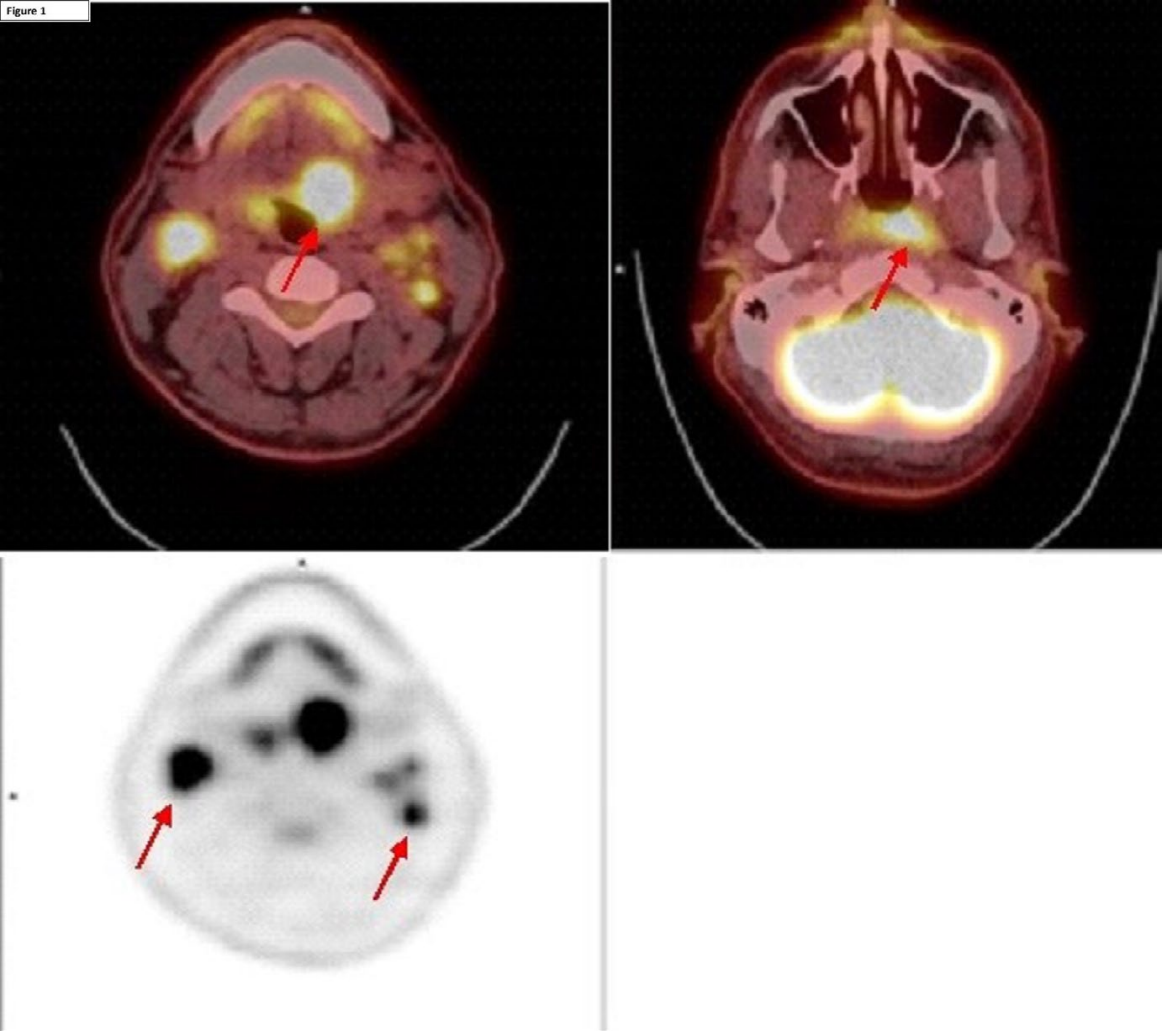
Axial section PET CT (top left), intense FDG uptake within tongue base mass (top right) higher FDG uptake on the left side of nasopharynx (bottom), and bilateral FDG avid cervical nodal metastases.

**FIGURE 2:**
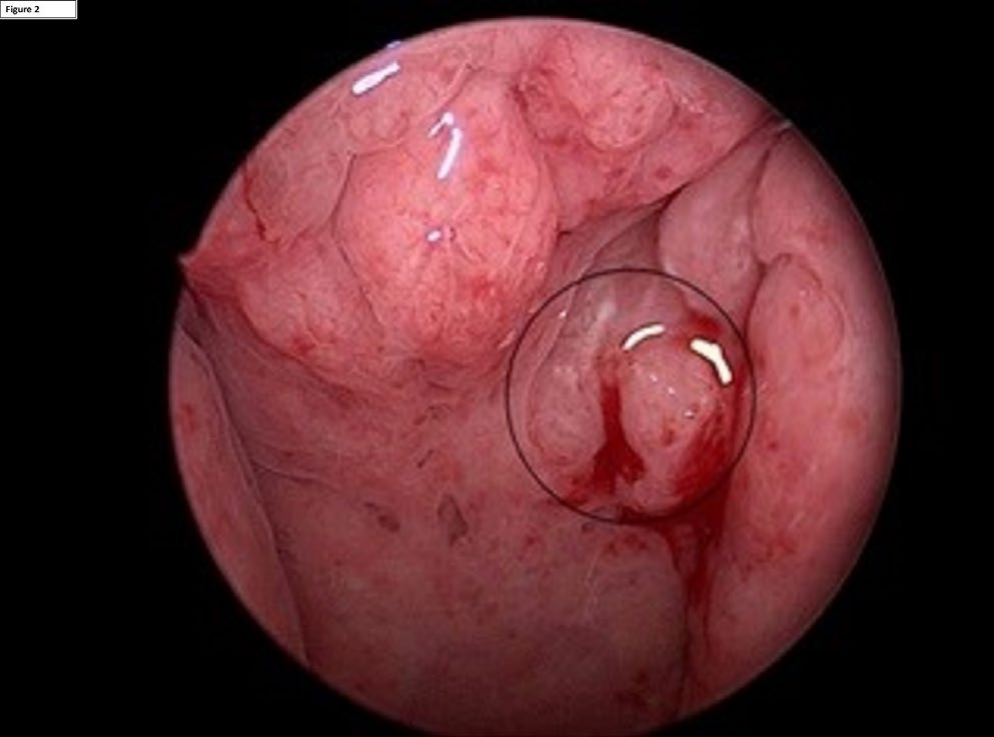
Endoscopic view of nasopharynx: fullness in the left FoR with prominent midline adenoid tissue.

**FIGURE 3:**
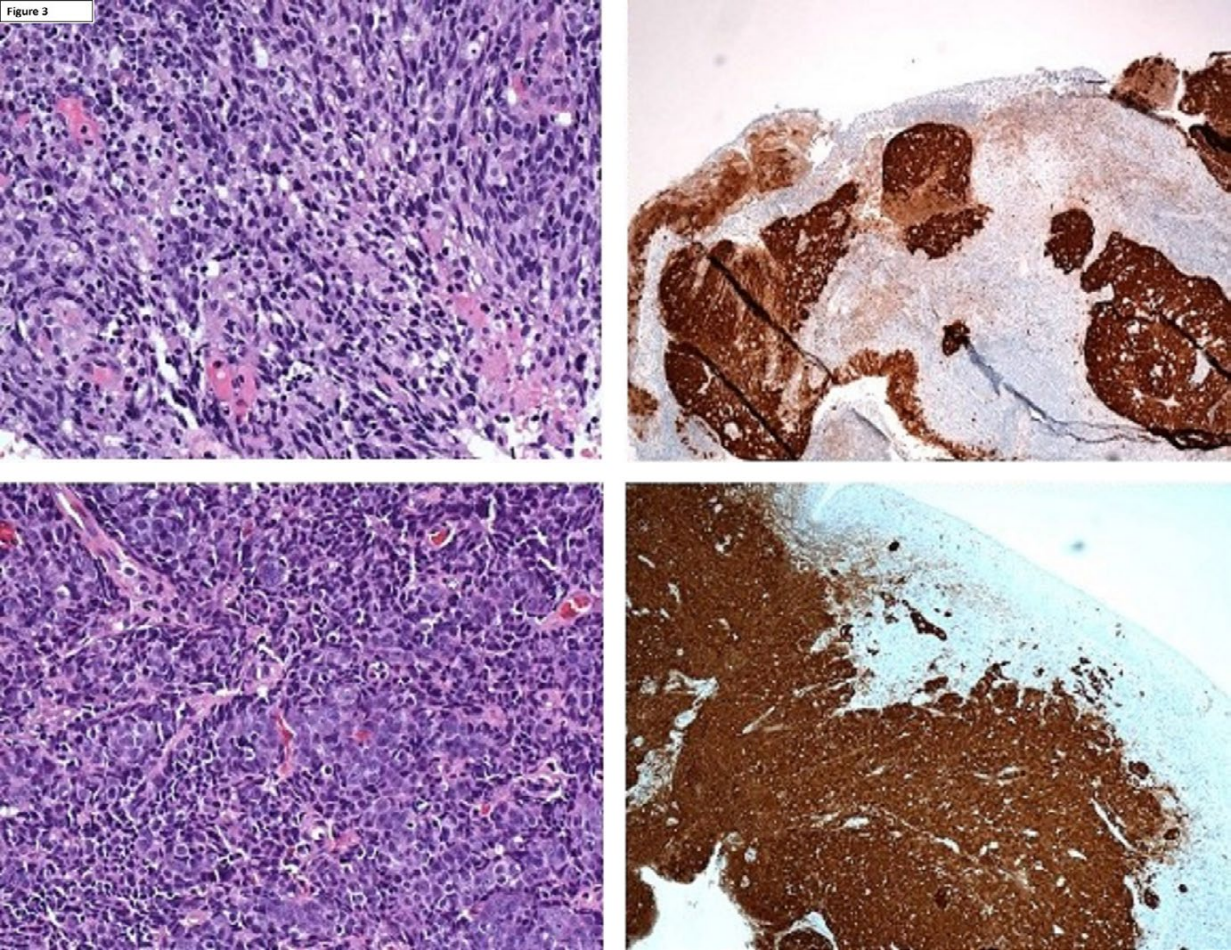
p16^+^ NPC (top) and OPC (bottom). (Top left) Nonkeratinizing SCC (top right) P16 IHC positive in tumor (bottom left) atypical scattered cells with dense lymphoid infiltrate (bottom right) IHC p16‐positive in tumor.

A 42‐year‐old Caucasian male presented with 2 months of left‐sided otalgia, odynophagia, and increasing left‐sided lymphadenopathy. He had a 25 pack year smoking history. Initial FDG‐PET/CT scan showed increased avidity in the left BOT and within multiple bilateral neck nodes. There was also increased FDG uptake within the left fossa of rosenmuller (FoR; Figure 1). MR imaging showed only mild asymmetry in FoR without a definite structural abnormality. Panendoscopy revealed a 4cm ipsilateral left BOT mass and fullness in the left FoR with prominent midline adenoid tissue (Figure 2). Initial biopsy was negative for malignancy at the left BOT and positive for a p16^+^/EBV^‐^ SCC from the nasopharynx (Figure 3). In the setting of an obvious BOT lesion, there was a concern regarding mislabelling and a second panendoscopy with biopsies demonstrated a p16^+^ SCC of the left BOT and normal nasopharyngeal tissue. A repeat PET/CT revealed ongoing avidity in the BOT and neck nodes, with reduced uptake in the nasopharynx (SUV max 6.15 vs initial SUV max 11.51). A repeat panendoscopy with nasopharyngeal adenoidectomy and deep biopsies of the left FoR confirmed the diagnosis of p16^+^/EBV^−^ NPC.

**FIGURE 4:**
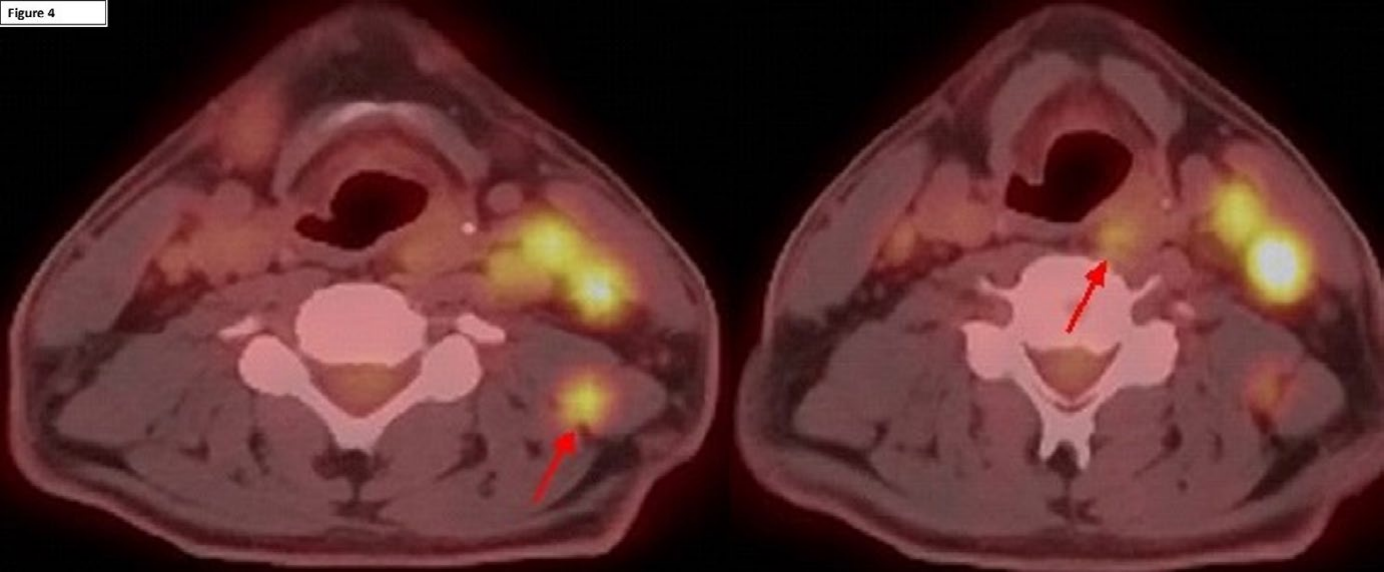
PET/CT (left half) Intense FDG avid node deep to left levator scapulae. (Right) Moderate FDG avid left parapharyngeal node at level of hypopharynx.

**FIGURE 5:**
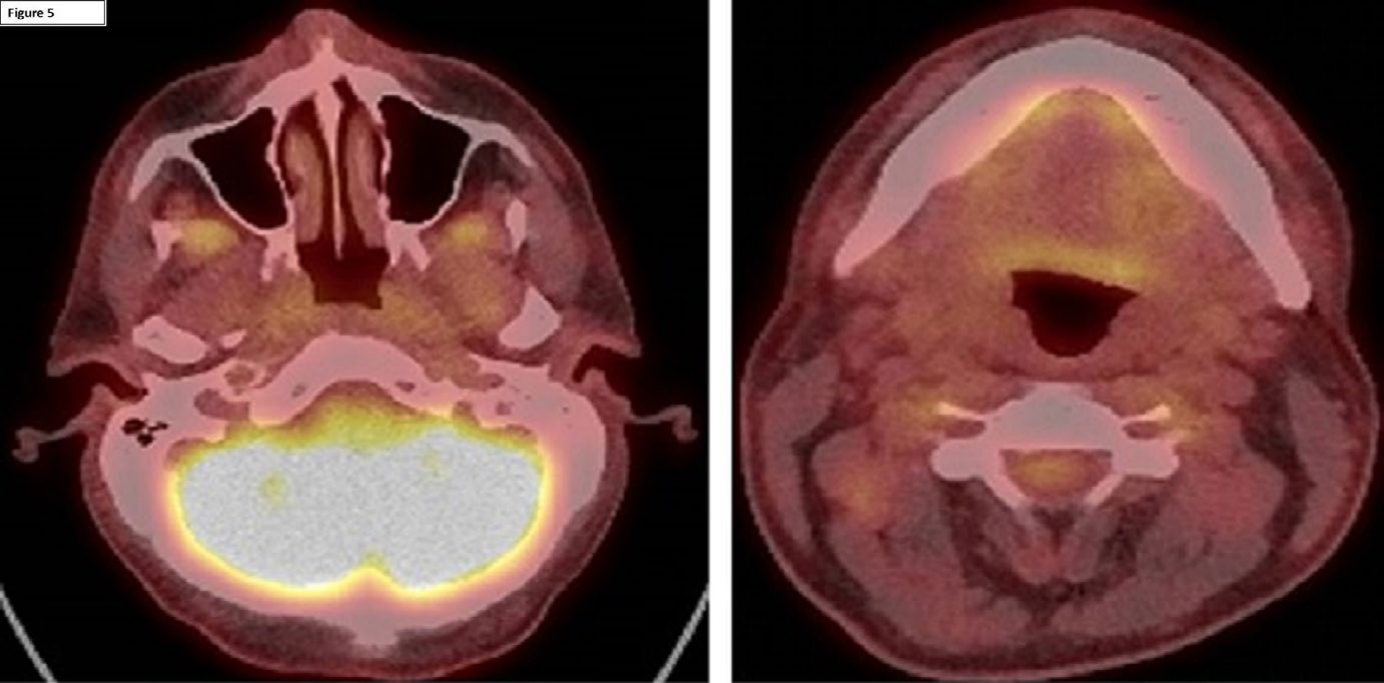
Three months post‐treatment, PET/CT with complete metabolic response.

Final clinical staging was T3 OPC and T1 NPC with bilateral neck nodal involvement (N2). Noteworthy in this case was the location of two nodal deposits identified on both PET/CT and cross‐sectional imaging which were located outside the usual prophylactic clinical target volumes (Figure 4): (a) a node deep to levator scapulae and (b) a parapharyngeal node at the level of the hypopharynx. A recommendation was made for radical intent chemoradiotherapy, 70Gy in 35 fractions to both primaries, and bilateral necks with concomitant three‐weekly cisplatin (100 mg/m^2^). Following percutaneous endoscopic gastrostomy insertion (PEG), treatment was completed without a break and with the anticipated acute treatment toxicities. PET/CT 3 months post‐treatment confirmed complete metabolic response (Figure 5). On last review (3 months post‐treatment), he remains PEG dependent able to swallow small amounts of thin fluids.

## 3 DISCUSSION

We report a case of synchronous p16^+^ SCC involving the BOT and nasopharynx. To our knowledge, this is one of only two reports of synchronous HPV‐associated SCC involving the oropharynx and nasopharynx.^3^ There are, however, reports of metachronous p16^+^/HPV^+^ NPC diagnosed following prior OPC treatment, raising the possibility of occult primaries lying within the nasopharynx in these cases.^2^

This case presented a diagnostic dilemma. There was not uniform agreement at our multi‐disciplinary meeting, and it was questioned whether the initial biopsies had been mislabelled given the panendoscopy (and negative biopsy) and MR findings of an obvious tumor in the BOT. Although the FDG‐PET/CT showed an abnormality in the nasopharynx, there was no corresponding structural abnormality and false‐positive FDG‐PET/CT findings in the head and neck are well documented.^4^ The repeat panendoscopy and PET/CT with corresponding reduction in SUV further challenged the presence of a nasopharyngeal primary, but nasopharyngeal adenoidectomy was felt to be prudent given a degree of uncertainty, which confirmed a synchronous p16^+^ NPC.

While treatment of the nasopharynx was possible from the outset, treating both the BOT and nasopharynx without sufficient proof will almost certainly subject a patient to excessive long‐term toxicities, including lifelong dysphagia and possible feeding tube dependence. Indeed, in this case a mean dose of 62Gy was delivered to the pharyngeal constrictors which is associated with higher chances of long‐term swallowing problems.^5^ In contrast, failing to treat the nasopharynx at the outset would likely lead to an unsalvageable recurrence. Just like the proposed recommendation by McGovern et al,^3^ we also recommend care full physical examination and biopsies in case of increased uptake on PET scan. While synchronous or metachronous p16^+^ OPC and NPC are rare, it is also possible that the elective coverage of level two, retropharyngeal and retrostyloid nodes will result in a dose wash through the nasopharynx which might be sufficient to eradicate microscopic disease.

International consensus guidelines on radiotherapy target volumes exist for both NPC and OPC. There are, however, no guidelines on the optimal management of synchronous p16^+^ primary HNC. While OPC cases are typically treated with isometric expansions (5 + 5 mm) from gross disease with elective coverage of nodal volumes, NPC treatment paradigms are largely based on outcomes from EBV^+^ cases, mandating widespread elective coverage of the base of skull. Whether this wide‐field coverage is necessary in HPV^+^ NPC cases is not currently known, but warrants further investigations.

In conclusion, we reported a unique case of synchronous p16^+^ SCC involving both the BOT and nasopharynx. In the setting of a relatively normal appearing nasopharynx on MRI, PET/CT may be useful in detecting occult p16^+^ SCC in the nasopharynx and should guide more extensive sampling, which may include adenoidectomy in selected patients.

## CONFLICT OF INTEREST

None declared.

## AUTHOR CONTRIBUTION

Muhammad Ali: design of concept and writing initial draft. Hock Kua: contributed in provision, citation of Figure 3 and revision of draft. Charlie Giddings: provision and citation of Figure 3, contributed in revision of draft. Daphne Day: concept design and revision of draft. Lachlan McDowell: final revision of draft before publication.

## Institutional review Board Statement

In line with our institutional REB policy, ethical clearance was not required for this case report. However, patient consent was obtained for publication of details, including images, provided in this report.
